# The open toolbox for behavioral research

**DOI:** 10.3758/s13428-023-02199-x

**Published:** 2023-10-04

**Authors:** Tobias Otto, Jonas Rose

**Affiliations:** 1 Cognitive Psychology, Faculty of Psychology, Ruhr University Bochum, Universitätsstraße 150, 44780 Bochum, Germany; 2 Neural Basis of Learning, Faculty of Psychology, Ruhr University Bochum, Universitätsstraße 150, 44780 Bochum, Germany

**Keywords:** MATLAB, Network, Stimulus presentation, Digital IO

## Abstract

In this work, we describe a new open-source MATLAB toolbox for the control of behavioral experiments. The toolbox caters to very different types of experiments in different species, and with different underlying hardware. Typical examples are operant chambers in animals, with or without neurophysiology, behavioral experiments in human subjects, and neurophysiological recordings in humans such as EEG and fMRI. In addition, the toolbox supports communication via Ethernet to either control and monitor one or several experimental setups remotely or to implement distributed paradigms across different computers. This flexibility is possible, since the toolbox supports a wide range of hardware, some of which is custom developments. An example is a fast network-based digital-IO device for the communication with experimental hardware such as feeders or triggers in neurophysiological setups. We also included functions for online video analysis allowing paradigms to be contingent on responses to a screen, the head movement of a bird in an operant chamber, or the physical location of an animal in an open arena. While the toolbox is well tested and many components of it have been in use for many years, we do not see it as a finished product but rather a continuing development with a focus on easy extendibility and customization.

## Introduction

Here we introduce a new MATLAB project, the Open Toolbox for Behavioral Research (OTBR). The primary application of the system is to control behavioral experiments in a wide range of setups and species. It offers a one-stop solution to implement feeding, visual or acoustic stimulation, camera tracking integration with MRI or physiology hardware, and many more. All hardware specifications are provided in one place (myHardwareSetup.m) such that experiments are easily portable between different hardware. The applications created with the OTBR can be individually controlled by a PC or configured as racks of multiple clients with a central control PC. The software can either be used on regular computers or on Raspberry Pi microcomputers without changing the experiments. Several computers running OTBR can be connected via WiFi or LAN to create larger, distributed setups. This versatility makes the toolbox very useful for technicians responsible for different labs with diverse research portfolio since it can serve as a one-stop solution for a wide range of different projects. The aim of this manuscript is to give an idea of the capabilities and the design of the toolbox, extensive documentation is beyond the scope of this manuscript and is therefore provided online (https://gitlab.ruhr-uni-bochum.de/ikn/OTBR/-/wikis/home).

The software platform is programmed in MATLAB as an open toolbox such that complete paradigms or individual functions can be used and shared. While MATLAB itself is neither open source nor freely available, our toolbox generally does not rely on any commercial MathWorks toolboxes and can therefore be used with the free and open-source alternative Octave (the few exceptions to this rule are clearly marked in the documentation). Octave is freely available for Windows, Mac, and Linux, and can offer a cost-effective solution should a MATLAB license be unavailable or impractical. We chose MATLAB even though it is commercial software since it is widely used in our field for both data analysis and for experimental control. Additionally, it offers good hardware support and functions, for instance for video-image processing. Therefore, MATLAB can provide an easy one-stop solution for experimental control and for data analysis that benefits a wide range of users and can eliminate the need to learn additional programming languages. In addition, MATLAB allows compiling experiments into a binary executable that can easily be shared and executed on machines without a full MATLAB installation and license. Consequentially, the OTBR supports compilation of entire experiments, which allows users to program and test their experiments in the laboratory and share the final program in a manner that is safe from modification and that can be run without knowledge of the software.

The OTBR uses the extensively adopted open-source Psychophysics toolbox (Brainard, 1997; Kleiner et al., 2007; Pelli, 1997), which offers a wide range of software tools and many useful extensions in its own regard. In many ways, this project is an expansion of our own Biopsychology toolbox, which has been used successfully in several laboratories and in a wide range of experiments (Rose et al., 2008). With the introduction of OTBR, we completely re-worked the underlying code, heavily expand on the capabilities of the Biopsychology toolbox, and include new hardware solutions to offer a complete system for behavioral experiments across different species. In no way do we view this platform as a finalized effort. Our goal is to share our own tools with the community and to encourage active participation in the project. Consequently, we welcome the contribution of compatible software, feature requests, bug reports, suggestions, or support requests. We aim to grow the toolbox to not just accompany our current experiments – the driving force behind our own developments – but to enable a wide range of species, experiments, and applications. Thus far, the toolbox is used with birds (pigeons, crows, quail, and jackdaws) rats and humans. In animals, it was used in studies of behavior, pharmacology, single-cell neurophysiology, optogenetic stimulation, and functional magnetic resonance imaging (fMRI) (Hahn et al., 2021; Horváth et al., 2016; Lukkes et al., 2016; Packheiser et al., 2021; Rook et al., 2020, 2021; Sonntag et al., 2014). In humans, the toolbox was used to conduct questionnaires, computer-based paradigms, measure electrodermal activity (EDA), electroencephalogram (EEG), fMRI, and to perform eye tracking (Bamberg et al., 2021; Beck et al., 2022; Hagedorn et al., 2022; Kinner et al., 2017; Schwabe & Wolf, 2009; Zöllner et al., 2022). With the aim of creating a one-stop solution for a wide range of experiments in mind, OTBR supports different hardware and can be used to control different types of setups. A major novelty of our toolbox is the compatibility with a variety of hardware, be it different digital input-output (DIO) devices or to run entire paradigms on Raspberry Pi. The latter is a great hardware platform since it offers sufficient computing power for most behavioral paradigms, it directly provides IO-pins for the control of external hardware such as feeders, and it is small and highly affordable, giving great scalability to training setups. By heavily relying on Ethernet or WiFi for communication, the platform can cater to very different use cases as unusual as distributed setups with multiple Raspberry Pi presenting stimuli and monitoring responses in different locations. Importantly, the same software can be run on the different supported setups (Windows, Mac, and Linux) without much customization.

## Outline of the toolbox

**Fig. 1:**
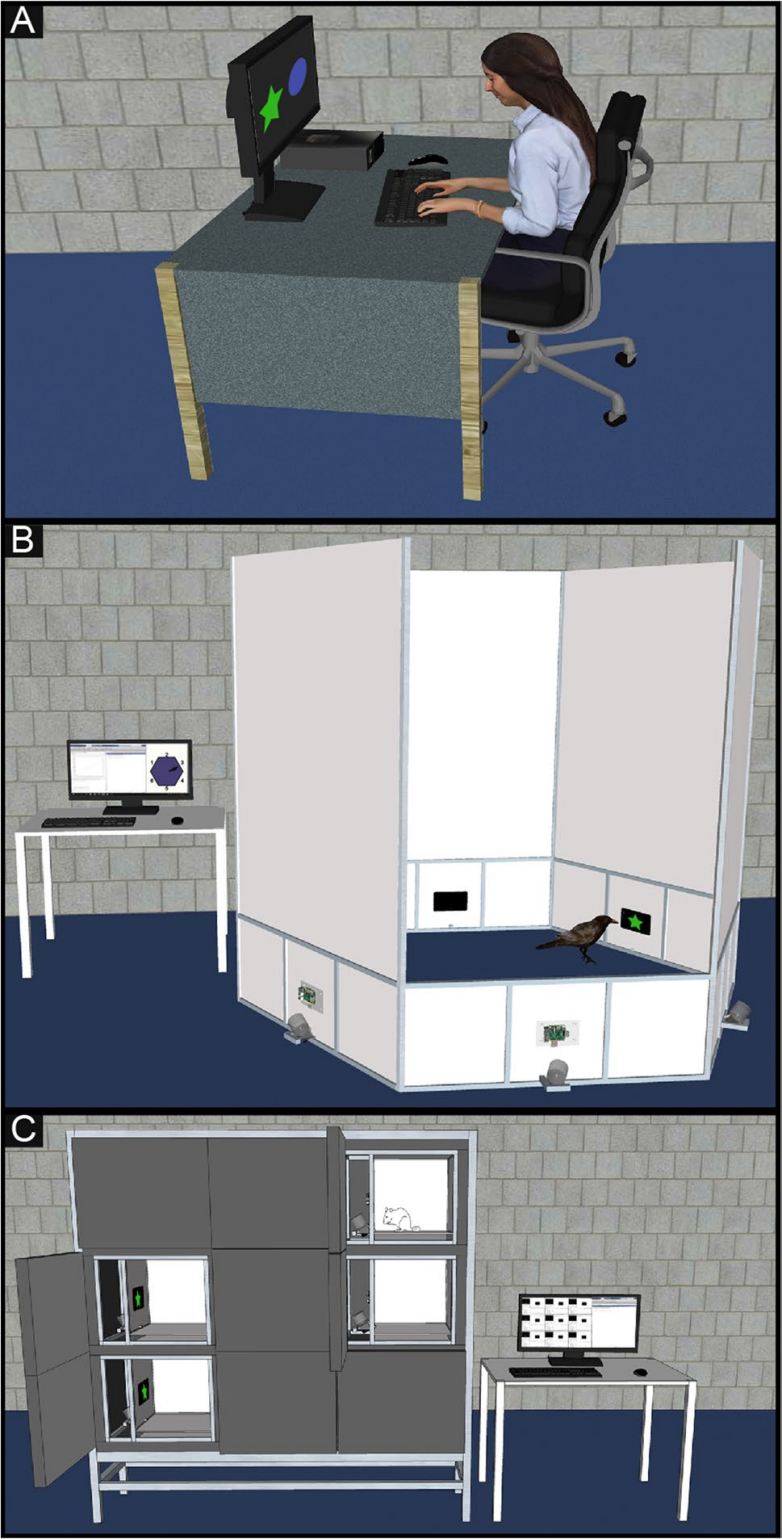
Typical use-cases. **A** The toolbox can run on a single computer. We use this configuration to develop and test paradigms, to conduct human behavioral, EEG, or MRI studies or to control one operant chamber. This configuration is useful for graphics-intense or timing-critical applications. **B** Multiple operant-modules are controlled by a single PC to run a distributed paradigm. Here we illustrate this with a hexagonal arena that is used to show stimuli, wait for responses, and deliver rewards at different locations in the arena. The paradigm is executed on the control-PC and individual commands or code-blocks are sent to and executed on the operant-modules. **C** A single PC is used to control multiple operant chambers. Here the operant-module in each chamber runs an experiment independently. One central computer is used to start and monitor the local code-execution. This configuration can be used to train multiple animals in parallel. In our example, the operant chambers are positioned in a rack and controlled by an adjacent computer. Since the communication is Ethernet-based, setups do not need to be placed in the same room as the control computer

In this section, we provide an outline of the project along with use cases that are typical in our work (Fig. 1). However, in no way do we wish to imply that potential applications of the platform are limited to these examples. Instead, we hope to highlight the flexibility of the platform and its ability to function in a wide range of scenarios. In fact, the software (and hardware) as we describe it here should be understood as an example or ‘teaser’ of a larger project. We believe that you know best where this approach could benefit your own experiments and that you should adopt the tools as needed and, hopefully, extend the toolbox. Some code examples for the purpose of illustration are included in Box 1, for detailed documentation and schematics, please refer to the online presence of the project (Downloads: https://gitlab.ruhr-uni-bochum.de/ikn/OTBR, Wiki: https://gitlab.ruhr-uni-bochum.de/ikn/OTBR/-/wikis/home). 

The software is packaged as a MATLAB toolbox with functions for randomization, visual stimulus presentation on monitors, auditory stimulation, and hardware interaction including trigger handling. The toolbox can address several digital IO (DIO) devices and it can easily be adapted to different devices – MATLAB offers support for many commercial systems, making the addition of new hardware to the toolbox particularly easy. Importantly, the software is platform-independent, and we currently use the toolbox on Windows and Linux systems as well as Raspberry Pi microcomputers where it runs under Octave instead of MATLAB. We also provide functions to monitor and control experiments remotely through an Ethernet connection. This feature also allows controlling experiments on different setups from a central machine or running paradigms on different computers simultaneously.

**Fig. 2:**
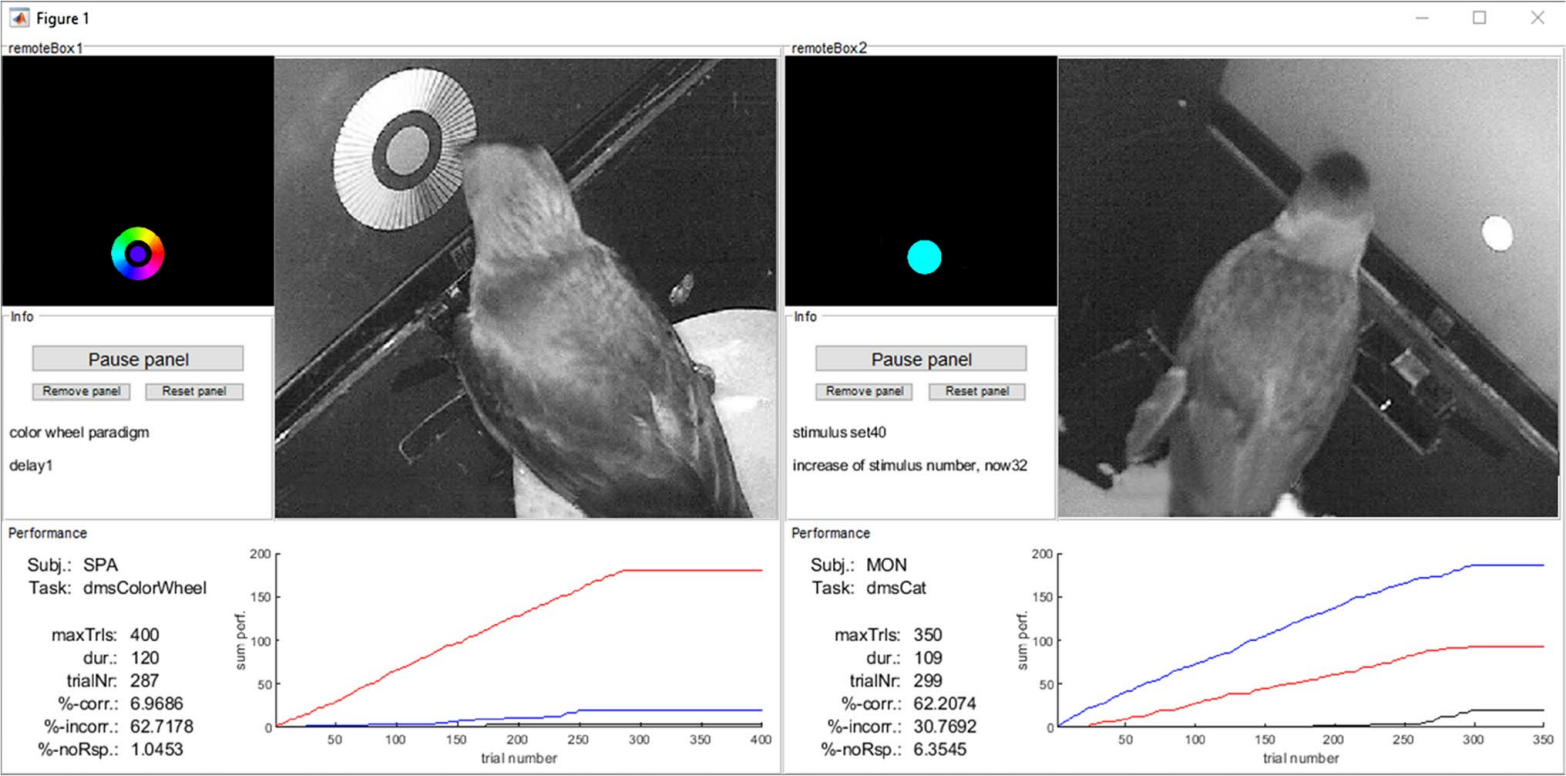
An example use of clientPanels (PC-screenshot). Here two clientPanels are added to one MATLAB-Figure to monitor two experiments remotely. Several subPanels are selected in both cases that allow the integration of a webcam, a paradigm viewer that displays the stimuli currently presented in the setup and a progress plot to monitor the behavior of the subject. Clientpanel also allow to add panels to remotely control the execution of paradigms, for instance if a central computer is set to control a rack of training boxes

For Raspberry Pi, we offer a cloned operating system that can be copied onto a micro-SD card to serve as a hard drive of the microcomputer. The clone includes Octave, the psychophysics, and OTBR toolboxes, and you only need to adjust Ethernet settings to your local requirements. While there is no need to install the toolbox, most of the functions rely on initialization (initOTBR.m) and hardware definitions (myHardwareSetup.m, myParadigmSetup.m) within a given paradigm. By adjusting one file (myHardwareSetup.m), it is possible to freely transfer entire paradigms between different setups and sometimes even species. This makes it possible to not only run identical paradigms on different hardware (e.g., Raspberry Pi or PC) but it also allows to develop and test new paradigms in the office and then deploy the final paradigm to the experimental setup. In the office, one can use a keyboard or mouse to test a paradigm that relies on touchscreen inputs and digital outputs in the experimental setup. Likewise, an experiment can be copied over the network to an experimental computer to be started and monitored remotely through the toolbox – useful for instance if dedicated hardware must be tested or if subjects must be left undisturbed by the experimenter. We offer a graphical user interface for the remote monitoring and control of experiments. These clientPanels (Fig. 2) are modular figures composed of several subpanels to include, for example, the stream from an IP camera, a panel to receive updates of the experimental progress or a presentation of the stimuli displayed to the subject. A subpanel to load and start an experiment is also available. The modular nature of this approach allows us to easily add custom panels.

## Exemplary use cases

### Human behavior

For a simple behavioral experiment, only a computer with the OTBR toolbox, a monitor for stimulus presentation, and a keyboard or mouse for response monitoring are required (Fig. 1A). We often use more complex setups with multiple monitors or with monitors with high refresh rates (the toolbox was tested up to 240 Hz on a DELL Alienware AW2518HF). In addition, several functions allow for quick paradigm design such as functions for controlled stimulus pseudo randomization (randomOrder.m) and outcome monitoring. We also added functions dedicated to human subjects such as the display of questionnaires and the use of scales to query ratings.

### Human neurophysiology

The toolbox is also routinely used in experiments recording EDA, eye tracking, EEG, fMRI, or each. Such experiments require the time-critical transmission and recording of hardware triggers. To send or receive triggers within an experiment, a supported hardware device, such as our open-source LAN DIO device (Box 2), must be selected in only one place in the code (myHardwareSetup.m). To receive triggers only one more line of code is needed in the experiment and to send triggers only one line per event is necessary. Adding new DIO hardware to the toolbox is straightforward if the device is supported by MATLAB. The OTBR toolbox also provides functions that handle MRI specific procedures such as waiting with execution of the experiment until a certain number of hardware triggers occurred. If the fMRI triggers are transmitted via USB, usually no additional hardware is needed, and the experiments can be started directly.

### Operant chambers

A single computer runs the OTBR toolbox to control one operant chamber. Here we place a monitor inside the operant chamber for stimulus presentation and another monitor next to the setup to control the paradigm. Responses of the animals are registered either using a touchscreen monitor or by utilizing transparent response keys. Using our Ethernet-based-IO device (Box 2) we control lights and feeders, read response keys, and send synchronizing triggers to neurophysiology hardware.

We use this type of setup mostly when timing is critical, for example for neurophysiological recordings. The system also controls a custom pellet feeder for food delivery and a fast video-based tracking module (the only OTBR function that requires additional MathWorks toolboxes) that we use to reconstruct the gaze direction of crows in real time (currently up to 150 Hz, camera limitation). We can also use the tracking module, for example to train the animals to hold the head in a certain position on each trial. The tracking module can easily be modified; for example, we use the same system to replace touchscreen monitors or to track an animal’s location in an open arena where we control the experiment contingent on the animals’ location. The module supports multiple cameras simultaneously, used for instance in three-dimensional gaze tracking or when operating in a large open arena (currently with up to four cameras).

### Operant modules

To our knowledge, this is a completely novel approach for controlling behavioral experiments. We designed operant modules utilizing Raspberry Pi microcomputers. Operant modules are stand-alone units that consist of a Raspberry Pi, a touchscreen, our pellet feeder, and a simple circuit board for power and IO handling. Raspberry Pi computers have several advantages: They are readily available at a low cost, they have very small footprint, comparably low heat exhaustion, and they are equipped with an Ethernet port. The most important property for our application, however, is that Raspberry Pi are equipped with digital and analog IO ports, eliminating the need for additional IO cards. Hardware such as lights, feeders, etc., can be controlled directly from the Raspberry Pi by using our circuit board or other community solutions. Additionally, several monitors and touchscreens are available for use with Raspberry Pi, useful for stimulus presentation and as input device. By using Raspberry Pi, our operant modules offer highly flexible solutions for very different setups and experiments.

The Raspberry Pi of each module runs Linux, Octave, the Psychophysics toolbox and the OTBR toolbox. Each module can operate fully independently, acting like the computer in the traditional operant chamber (Buscher et al., 2020; Geissmann et al., 2017). A module can also be positioned, for example, in the home cage of the animals for enrichment, for pre-training, or for full training. If needed, the modules can be configured to boot directly into the paradigm without the need to attach a keyboard, mouse, or additional monitor to the Raspberry Pi. Here data can be stored on a USB key or on a Network drive. For a different approach, we take advantage of the Ethernet connection of the Raspberry Pi. Modules can be controlled remotely through the OTBR toolbox such that a remote computer acts as a controller for one or for several modules. We developed this solution mainly with two use cases in mind: either to run distributed paradigms on several modules simultaneously (Fig. 1A) or to test multiple subjects in parallel (rack of operant modules, Fig. 1B).

### Distributed paradigms on several operant modules

A novel use case is distributed paradigms (Fig. 1B). Here the paradigm is executed on a control computer that controls several behavioral modules remotely. The program on the controller therefore remotely coordinates stimulus presentation, reward delivery and behavioral responses on the different modules. We use this design in paradigms where animals are required to move between modules to give different responses to the stimuli displayed on the modules. The communication between controller and the modules is implemented such that the controller sends a MATLAB command to the module and triggers its remote execution on the module. This allows running any MATLAB command giving the user ample flexibility. One command could be used to present a single stimulus, to deliver a reward, or to execute a full stimulus-response-outcome sequence – using one line of code on the controller. In most paradigms, we copy preprogrammed code snippets (scripts) to a network share on the Raspberry Pi of each module at the start of the paradigm. During runtime, we then only trigger the execution of these scripts from the control computer. Functions to collect response and timing data from the module are also available. This is particularly convenient if such code snippets can be reused between paradigms.

### Rack of operant modules

A rack of operant modules can be assembled to provide a convenient space- and cost-effective setup to train or test many animals simultaneously (Fig. 1C). The paradigms are started remotely using a single control computer for the entire rack. However, in this case, the Raspberry Pi of each module executes the full paradigm independently. Once the program is started, the control computer could be disconnected without affecting the execution of the paradigm, albeit we use it to monitor the paradigm. The controller is connected via Ethernet, so it does not have to be placed directly adjacent to the rack. In fact, the controller could be placed in a different room to allow the experimenter to monitor several racks while working nearby and without disrupting the animals. A graphical user interface (Fig. 2) on the controller can be used to control each module and to start individual paradigms remotely. This control panel can also be used to monitor the progress and to view a webcam inside each module. This unique solution offers a scalable space- and cost-effective system for high-throughput behavioral testing.

## Synopsis

The OTBR toolbox offers an easy and highly flexible approach to programming behavioral and neurophysiological experiments in MATLAB. This toolbox is the expansion (and full re-development) of the Biopsychology toolbox that has been in continuous use over the last 14 years. To make the project readily available it is open source. Importantly, we put considerable effort into the documentation to make the toolbox accessible to anyone with some programming experience in MATLAB. The OTBR toolbox is already in use with several species and very different hardware, for instance different digital IO devices. A wide range of experiments ranging from fMRI and EEG experiments in human subjects to optogenetics in pigeons and neurophysiology in crows are implemented using the OTBR toolbox. Particularly, the LAN tools gave our work novel approaches. We can now construct comparably small and affordable setups for high-throughput testing. Importantly, we can also design completely new, distributed paradigms that take our research in new directions. For our laboratories, a great appeal is the ability conduct such a wide range of different experiments with a very similar code base. In many cases, experiments can be conveniently ported between entirely different setups and species with only minor modifications to a single hardware config file (myHardwareSetup.m). We aim to share this project with other laboratories in the hope to not only pass on the tools that we developed for our own work but also to gain further contributions to the toolbox.

**Box 1: Software examples**

The first stop for any potential use of novel code is always the documentation. Since we provide this elsewhere (https://gitlab.ruhr-uni-bochum.de/ikn/OTBR/-/wikis/home) along with a complete function reference, examples, and instructions on how to get started, it is well beyond the scope of this article. Instead, we chose to include only a few illustrations of OTBR functionality in allow the reader to get some sense of our approach.

## Questionnaires and scales for human subjects

A simple questionnaire (or a scale for ratings) can be generated with one line of code and executed with one simple command. The questionnaire responds to mouse or touchscreen input and reports all relevant information back. These functions are useful either to conduct full standalone experiments or, for instance, to collect additional information before an EEG.

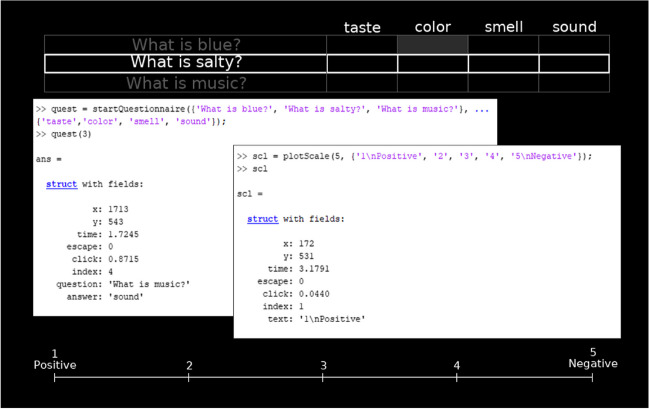



### Pseudo randomization

The function randomOrder.m can be used for stimulus randomization. The function generates pseudorandom sequences of values and is highly customizable. By default, it prevents the excessive accidental repetition of one value.

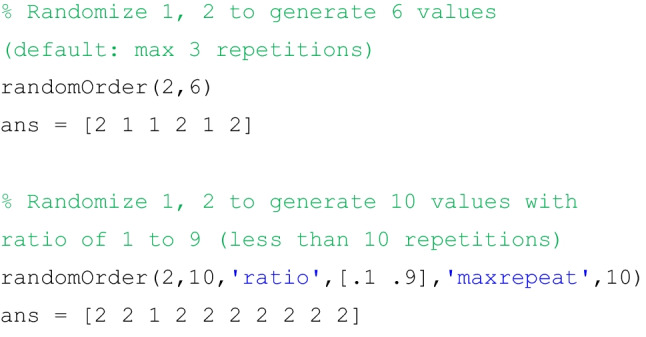



### Input monitoring

We use the function keyBuffer to halt program execution until a certain behavioral criterion is met. The function can either monitor response keys on the keyboard, the status of physical DIO pins, a touch, or a mouse click to a pre-defined response region on a monitor. We also use the same function to react to camera-based head tracking or whole animal tracking.

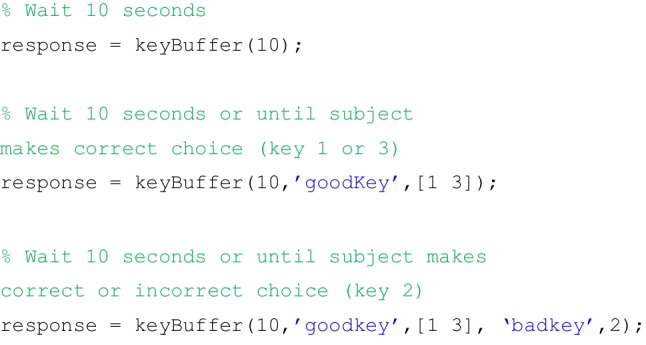



### Adapting to different underlying hardware

All hardware definitions are stored in one file (myHardwareSetup.m). This allows us to easily move a paradigm to a new setup or to propagate code changes to different hardware by simply copying all files except one. Furthermore, the necessary changes to transition between different supported hardware are very simple. For example, the underlying (supported) IO device can be switched using one line of code. 
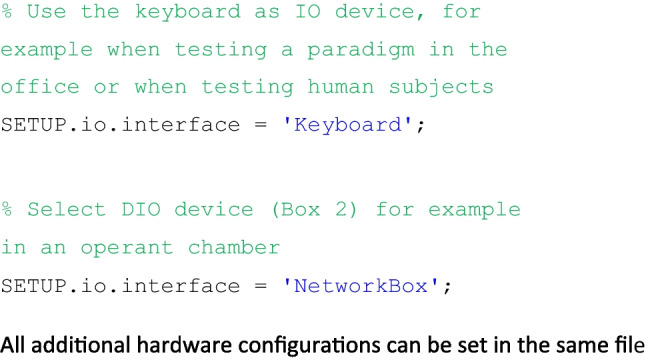

**Box 2: Digital IO over LAN**

The toolbox also supports a DIO device that we developed to communicate efficiently with different types of hardware. We chose Ethernet for its flexibility, availability, ease of use and, importantly, for its speed. On the hardware side, we decided to use a microcomputer board (Odroid C1+) that is comparable to Raspberry Pi but enables faster communication by offering a gigabit Ethernet connection. The principle of our approach is simple: the system is set up by coping a fully cloned (Linux) operating system onto a micro-SD or eMMC card that serves as the hard dive of the Gigabit- Ethernet-based DIO device. A DIO round-trip can be realized in under 250 μs. The first trace (*turquoise*) is the state-change of a digital pin on our DIO device. The toolbox is then used to read this signal back through another pin on the DIO device. Once the state-change is detected in the toolbox, it set another digital pin high. This is visible in the second trace (*magenta*). The lag between the two traces is the roundtrip time
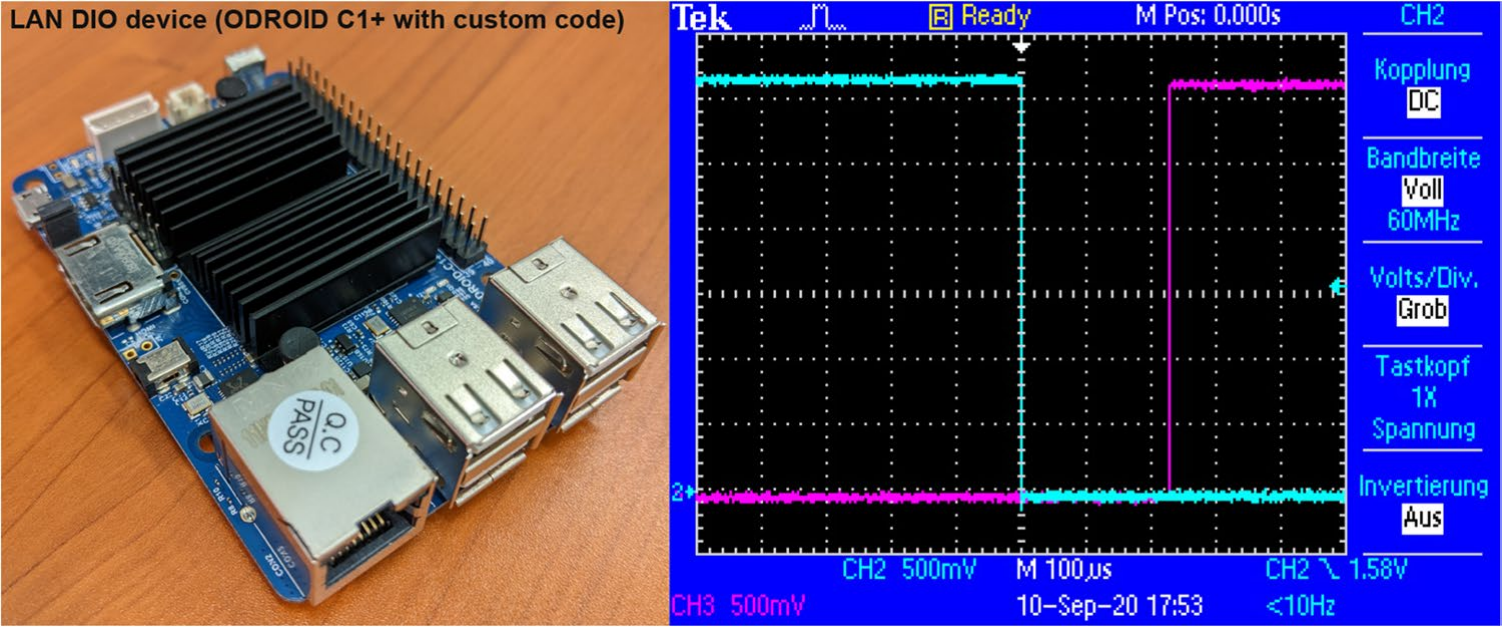
microcomputer. Only the Ethernet settings must be adjusted to the local requirements. After booting, the microcomputer awaits a data package over Ethernet. If such a package received, the state of the onboard output pins is changed according to the received data. In the same manner, if an input pin changes its value, the microcomputer sends a defined data package over Ethernet. This system is very fast, allowing a full roundtrip (write-read-write) in under 1 ms. Therefore, this device offers sufficiently low latencies to replace traditional PCI/PCIe IO devices even in demanding neurophysiological applications. Compared to internal cards, this new approach dramatically increases hardware flexibility since virtually any type of computer (including laptops) can be used without modification or the use of proprietary hardware, cables, etc. Ethernet is one of the most abundant, tested, and flexible communications systems between computers. If speed is not critical, a Raspberry PI can be used with the installation of the OTBR toolbox instead of the ODROID.

## Data Availability

The work outlined in this manuscript is the development of a new software tool. All code is made publicly available, documented extensively, and published under an open-source license. No relevant research data were obtained.
